# High Prevalence of Artemisinin-Resistant *Plasmodium falciparum*, Southeastern Sudan

**DOI:** 10.3201/eid3106.241810

**Published:** 2025-06

**Authors:** Mariangela L’Episcopia, Albadawi A. Talha, Bakri Y.M. Nour, Ibrahim Mohamed Ahmed Sana, Emmanuelle Caspar, Lucas Thiebaut, Lucien Platon, Buze Chala, Laurence Ma, Lemu Golassa, Edvige Perrotti, Carlo Severini, Didier Menard

**Affiliations:** Istituto superiore di sanità, Rome, Italy (M. L’Episcopia, E. Perrotti, C. Severini); Jouf University, Sakaka, Saudi Arabia (A.A. Talha); University of Gezira, Wad Medani, Sudan (B.Y.M. Nour, I.M.A. Sana); University of Strasbourg, Strasbourg, France (E. Caspar, L. Thiebaut, L. Platon, D. Menard); Addis Ababa University, Addis Ababa, Ethiopia (B. Chala, L. Golassa); Institut Pasteur, Paris, France (L. Ma, D. Menard); Centre Hospitalier Universitaire de Strasbourg, Strasbourg, France (D. Menard); Institut Universitaire de France, Paris (D. Menard)

**Keywords:** *Plasmodium falciparum*, antimicrobial resistance, Artemisinin, parasites, Sudan

## Abstract

We conducted a hospital-based cross-sectional study of *Plasmodium falciparum* in 2017 in southeastern Sudan. Among 257 *P. falciparum* samples, we found 22% harbored the *pfkelch13* R622I mutation and 10.7% showed *hrp2/3* gene deletions. Our findings highlight the urgent need for enhanced surveillance of drug- and diagnostic-resistant parasites in the Horn of Africa.

Malaria challenges global public health, especially in high-transmission areas of sub-Saharan Africa (*1*). Artemisinin-based drug combination therapy (ACT) has greatly reduced malaria illness and death, but the emergence and spread of partial resistance to artemisinin drug derivatives (ART-R) in *Plasmodium falciparum* threatens those gains. ART-R, which delays parasite clearance after 3 days of ACT, is associated with specific nonsynonymous polymorphisms in the propeller domain of the *P. falciparum kelch13* (*Pfkelch13*) gene (*2*–*4*). Although ACTs remain effective in Africa, recent reports have identified *Pfkelch13* mutations (R561H, C469Y, A675V, and R622I) linked to ART-R in several countries, particularly Rwanda, Uganda, Tanzania, and Eritrea (*5*–*9*). In addition, *P. falciparum* isolates with *hrp2/3* gene deletions, which evade detection by HRP2-based rapid diagnostic tests (RDTs), have emerged in the Horn of Africa, highlighting the need for vigilant monitoring of resistance and gene deletions (*8*,*10*).

Sudan, a major contributor to malaria cases in the World Health Organization Eastern Mediterranean Region (*1*), has struggled to meet the 2030 Global Technical Strategy targets. During 2015–2020, malaria incidence rose by >40%, leading to the adoption of the high burden to high impact strategy in 2022 (*11*). *P. falciparum* drug resistance, particularly to artesunate/sulfadoxine and pyrimethamine drugs, complicates those efforts, prompting a shift to artemether/lumefantrine drugs as first-line treatment. In addition, *hrp2* gene deletions were recently identified in >10% of *P. falciparum* isolates (*12*) (Appendix). In this study, we analyzed *P. falciparum* blood sample isolates from southeastern Sudan for molecular markers associated with antimalarial drug resistance and *hrp2* and *hrp3* gene deletions.

## The Study

We conducted a hospital-based cross-sectional observational study during August–December 2017 in Al Jazirah and Al Qadarif states, located in the east-central region of Sudan. Those sites are characterized by a subequatorial climate with a rainy season typically occurring during June–early November (*13*) (Appendix). All patients exhibiting clinical symptoms suggestive of malaria were eligible for blood sampling, after informed consent was obtained from the patient or parents. We confirmed *P. falciparum* malaria diagnosis through microscopy by using thick and thin blood films. Patients received treatment with AL drugs in accordance with the national drug policy. We spotted finger-prick blood onto Whatman 3MM filter papers (Whatman International, https://www.cytivalifesciences.com) to make dried blood spots for molecular analysis. The study received ethical approval from the Ministry of Health in Gezira State (approval no. MU/2019).

We extracted genomic DNA from three 3-mm punches of dried blood spots, as previously described (*14*). We conducted Illumina paired-end sequencing (Illumina, https://www.illumina.com) and selective amplification of parasite DNA to identify single-nucleotide polymorphisms in the *Pfkelch13*, *Pfcrt*, *Pfmdr-1*, *dhfr*, and *dhps* genes, as previously described (*8*). In addition, we assessed deletions of the *hrp2* or *hrp3* genes, which can lead to false-negative results in HRP2-based RDTs. We used laboratory reference parasite strains (Dd2, 7G8, HB3, and Cambodia culture-adapted strains) with known alleles and the presence or absence of *hrp2* and *hrp3* deletions as controls (Appendix).

We collected a total of 257 blood samples from 2 study sites: 170 from Al Jazirah and 87 from Al Qadarif. Demographic data were missing for 2 samples, resulting in a study population of 255 participants, 128 female and 127 male, ages 1–44 years. Most enrolled patients were febrile (95.6%), and their clinical manifestations were common to malaria, such as headache and vomiting (58.6%). After we examined the thick and thin blood films, we found all the patients were positive for *P. falciparum*.

Molecular analysis of the 257 samples revealed a high prevalence of the *Pfkelch13* 622I mutant parasites (21.8%, n = 56), a mutation validated by the World Health Organization as associated with ART-R. Among them, we identified 192 *Pfkelch13* wild-type, 52 *Pfkelch13* 622I single mutants, 1 *Pfkelch13* 494I single mutant, 7 *Pfkelch13* 625R single mutants, and 5 isolates with polyclonal infections (3 *Pfkelch13* 622I/625R, 1 *Pfkelch13* 622I/494I, and 1 *Pfkelch13* 494I/658T) (Table 1).

**Table 1:** Frequency of the *Pfkelch13*, *Pfcrt*, *Pfmdr-1*, *dhfr*, and *dhps* genotypes in blood samples collected in patients with *Plasmodium falciparum* malaria before artemisinin-based combination therapy from Al Jazirah and Al Qadarif, Sudan, 2017*

Gene	Codon	Amino acid	No. (%) samples			p value
			Al Jazirah	Al Qadarif	Total	
No. samples	NA	NA	170	87	257	NA
*Pfkelch13*†	Wild-type	NA	123 (72.4)	69 (79.3)	192 (74.7)	0.01
	494	Valine → Isoleucine	1 (0.6)	0	1 (0.4)	
	622	Arginine → Isoleucine	42 (24.7)	10 (11.5)	52 (20.2)	
	625	Glycine → Arginine	1 (0.6)	6 (6.9)	7 (2.7)	
	622/625	Arginine → Isoleucine / Glycine → Arginine	1 (0.6)	2 (2.3)	3 (1.2)	
	622/494	Arginine → Isoleucine / Valine → Isoleucine	1 (0.6)	0	1 (0.4)	
	494/658	Valine → Isoleucine / Lysine → Threonine	1 (0.6)	0	1 (0.4)	
*Pfcrt*	72	Cysteine	170 (100)	87 (100)	257 (100)	NA
		Serine	NA	NA	NA	
	74	Methionine	124 (73)	44 (51)	168 (65)	0.0004
		Isoleucine	46 (27)	43 (49)	89 (35)	
	75	Asparagine	124 (73)	44 (51)	168 (65)	0.0004
		Glutamic acid	46 (27)	43 (49)	89 (35)	
	76	Lysine	124 (73)	44 (51)	168 (65)	0.0004
		Threonine	46 (27)	43 (49)	89 (35)	
	93	Threonine	170 (100)	86 (99)	256 (99.6)	0.2
		Serine	0	1 (1)	1 (0.4)	
	356	Isoleucine	169 (99)	85 (98)	254 (98.8)	0.2
		Threonine	1 (1)	2 (2)	3 (1.2)	
*Pfmdr-1*	86	Asparagine	145 (85)	75 (86)	220 (86)	0.8
		Tyrosine	25 (15)	12 (14)	37 (14)	
	184	Tyrosine	20 (12)	14 (16)	34 (13)	0.3
		Phenylalanine	150 (88)	73 (84)	223 (87)	
	1034	Serine	139 (82)	70 (80)	209 (81)	0.8
		Cysteine	31 (18)	17 (20)	48 (19)	
	1042	Asparagine	170 (100)	87 (100)	257 (100)	NA
		Aspartic acid	NA	NA	NA	
	1246	Aspartic acid	170 (100)	87 (100)	257 (100)	NA
		Tyrosine	NA	NA	NA	
*dhfr*	51	Asparagine	79 (46)	16 (18)	95 (37)	<0.0001
		Isoleucine	91 (54)	71 (82)	162 (63)	
	59	Cysteine	151 (90)	76 (87)	227 (89)	0.4
		Arginine	16 (10)	11 (13)	27 (11)	
	108	Serine	72 (42)	22 (25)	94 (37)	0.007
		Asparagine	98 (58)	65 (75)	163 (63)	
	164	Isoleucine	170 (100)	87 (100)	257 (100)	NA
		Leucine	NA	NA	NA	
*dhps*	431	Isoleucine	170 (100)	87 (100)	257 (100)	NA
		Valine	NA	NA	NA	
	436	Serine	167 (98)	83 (95)	250 (97)	0.2
		Alanine	3 (2)	4 (5)	7 (3)	
	437	Alanine	30 (18)	35 (40)	65 (25)	0.0001
		Glycine	140 (82)	52 (60)	192 (75)	
	540	Lysine	162 (95)	72 (83)	234 (91)	0.0009
		Glutamic acid	8 (5)	15 (17)	23 (9)	
	581	Alanine	170 (100)	87 (100)	257 (100)	NA
		Glycine	NA	NA	NA	
	613	Alanine	169 (99)	86 (99)	255 (99)	0.6
		Serine	1 (1)	1 (1)	2 (1)	

*p values indicate the difference in percentages between sites. NA, not available. †*Pfkelch13* 622I/625R, 1 sample from Al Jazirah and 2 samples from Al Qadarif; *Pfkelch13* 494I/622I, 1 sample from Al Jazirah; *Pfkelch13* 494I/658T, 1 sample from Al Jazirah.

We further explored the genetic profile of the *Pfkelch13* R622 wild-type parasites from Sudan and 622I mutants at known antimalarial drug–resistance loci and the frequency of *hrp2* and *hrp3* deletions, a genomic feature previously observed in *Pfkelch13* 622I mutants in Eritrea and Ethiopia (*8*,*10*). We examined 201 *Pfkelch13* R622 wild-type and 56 *Pfkelch13* 622I mutant parasites for mutations across 4 genes (Table 2). Differences in allele frequencies were noted in the *Pfcrt* gene, associated with resistance to chloroquine, and the *dhfr* gene, associated with resistance to pyrimethamine. We conducted a stratified analysis that revealed a higher difference in *Pfcrt* allele frequencies in the Al Qadarif site compared with Al Jazirah. High proportions of *Pfkelch13* 622I parasites exhibited *Pfcrt* 74I/75E/76T/356T mutations (5.4% vs. 0% for *Pfkelch13* R622 wild-type parasites; p = 0.01) as well as the *dhfr* wild-type allele (41.1% vs. 18.9% for *Pfkelch13* R622 wild-type parasites; p = 0.001).

**Table 2:** Genetic backgrounds associated with antimalarial drug resistance and polymorphisms of *hrp2/3* genes of *Pfkelch13 R622* and *622I* isolates collected from patients with *Plasmodium falciparum* malaria before treatment from Al Jazirah and Al Qadarif, Sudan, 2017*

Gene	Allele		Al Jazirah		p value	Al Qadarif		p value	Total		p value
R622	622I	R622	622I	R622	622I
No. samples	NA		126	44	NA	75	12	NA	201	56	NA
*Pfcrt*	CVMNKTHFIMCGI	NA	93 (74)	31 (71)	0.2	39 (52)	5 (42)	0.005	132 (65.7)	36 (64.3)	0.01
	CVIETTHFIMCGI	74I/75E/76T	33 (26)	12 (27)		35 (47)	5 (42)		68 (33.8)	17 (30.4)	
	CVIETSHFIMCGI	74I/75E/76T/93S	0	0		1 (1)	0		1 (0.5)	0	
	CVIETTHFIMCGT	74I/75E/76T/356T	0	1 (2)		0	2 (17)		0	3 (5.4)	
*Pfmdr-1*	NYSND	NA	11 (9)	3 (7)	0.3	10 (13)	1 (8)	0.6	21 (10.4)	4 (7.1)	0.2
	YYSND	86Y	0	1 (2)		0	0		0	1 (1.8)	
	NFSND	184F	73 (58)	31 (70)		41(55)	9 (75)		114 (56.7)	40 (71.4)	
	NYCND	1034C	5 (4)	0		3 (4)	0		8 (4.0)	0	
	YFSND	86Y/184F	15 (12)	5 (11)		9 (12)	0		24 (11.9)	5 (8.9)	
	NFCND	184F/1034C	19 (15)	3 (7)		9 (12)	2 (17)		28 (13.9)	5 (8.9)	
	YFCND	86Y/184F/1034C	3 (2)	1 (2)		3 (4)	0		6 (3.0)	1 (1.8)	
*dhfr*	ACNCSI	NA	28 (22)	21 (48)	0.06	10 (13)	2 (17)	0.6	38 (18.9)	23 (41.1)	0.01
	ACICSI	51I	15 (12)	4 (9)		8 (11)	1 (8)		23 (14.4)	5 (8.9)	
	ACNCNI	108N	21 (17)	7 (16)		2 (3)	2 (17)		23 (14.4)	9 (16.1)	
	ACICNI	51I/108N	48 (38)	10 (23)		44 (59)	7 (58)		92 (45.8)	17 (30.4)	
	ACIRSI	51I/59R	4 (3)	0		1 (1)	0		5 (2.5)	0	
	ACNRNI	59R/108N	2 (2)	0		0	0		2 (1.0)	0	
	ACIRNI	51I/59R/108N	8 (6)	2 (5)		10 (13)	0		18 (9.0)	2 (3.6)	
*dhps*	SAKAA	NA	99 (79)	32 (73)	0.8	30 (40)	6 (50)	0.7	129 (64.2)	38 (67.9)	0.8
	SGKAA	437G	18 (14)	9 (20)		28 (37)	4 (33)		46 (22.9)	13 (23.2)	
	SAEAA	540E	6 (5)	2 (5)		14 (19)	1 (8)		20 (10.0)	3 (5.4)	
	SAKAS	613S	1 (1)	0		0	0		1 (0.5)	0	
	AGKAA	436A/437G	2 (2)	1 (2)		2 (3)	1 (8)		4 (2.0)	2 (3.6)	
	AAKAS	436A/613S	0	0		1 (1)	0		1 (0.5)	0	
*hrp*	no deletion	NA	112 (89)	35 (80)	0.1	56 (75)	7 (58)	0.3	168 (83.6)	42 (75.0)	0.09
	hrp2 deletion	NA	1 (1)	0		0	0		1 (0.5)	0	
	hrp3 deletion	NA	11 (9)	5 (11)		15 (20)	3 (25)		26 (12.9)	8 (14.3)	
	hrp2/3 deletion	NA	2 (2)	4 (9)		4 (5)	2		6 (3.0)	6 (10.7)	

*Values are no. (%) except as indicated. p values are the differences in proportions between *Pfkelch13 R622* and *Pfkelch13 622I* parasites. NA, not available.

We also analyzed *hrp2* and *hrp3* deletions. We found a higher proportion of *Pfkelch13* 622I parasites with both *hrp2* and *hrp3* deletions compared with *Pfkelch13* R622 wild-type parasites (10.7% vs. 3.0%; p = 0.02) (Table 2; Figure). This difference was significant in samples from Al Jazirah (p = 0.04).

**Figure:**
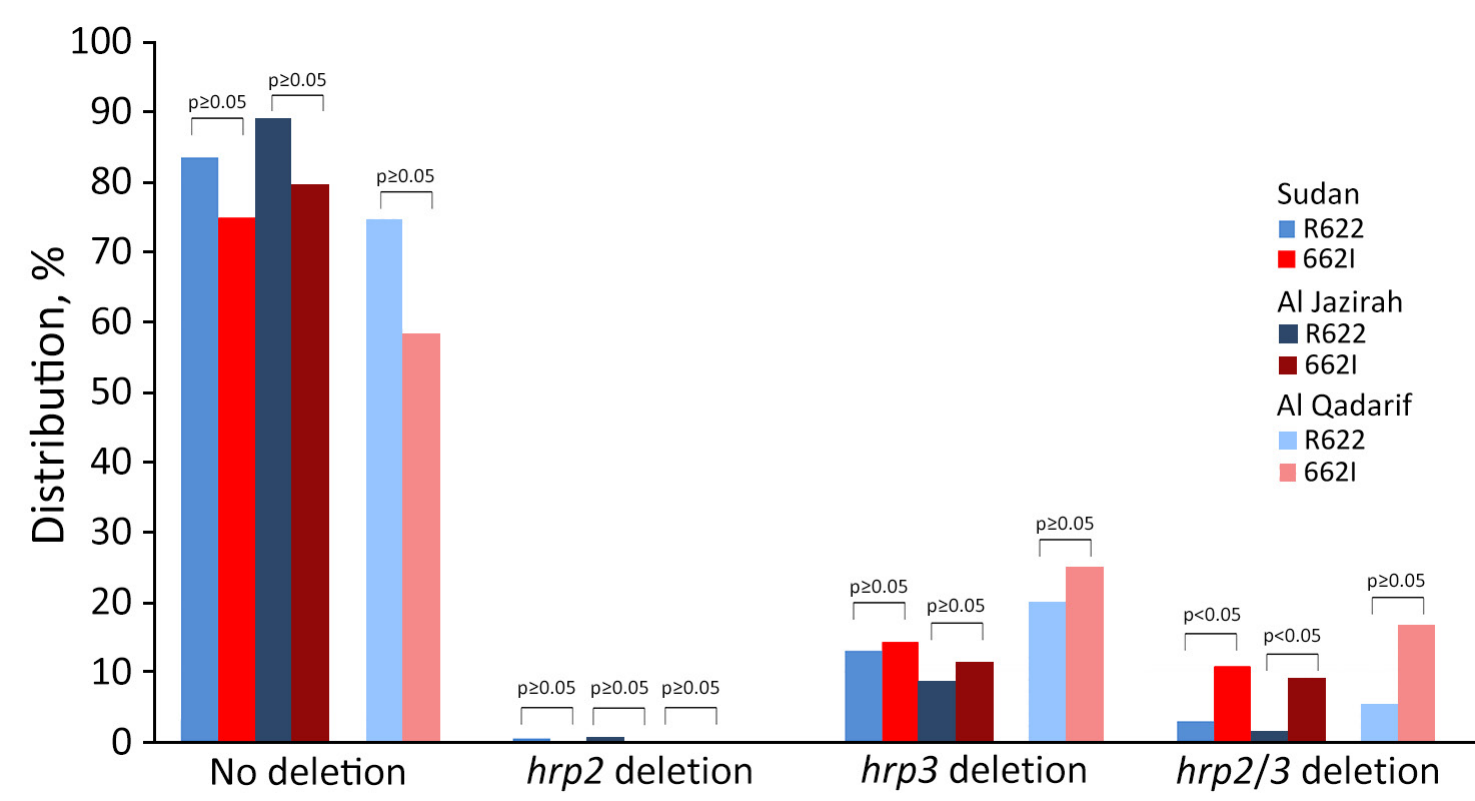
Distribution of artemisinin-resistant *Plasmodium falciparum*, southeastern Sudan, 2017. Percentages of *Pfkelch13* R622 and *Pfkelch13* 622I *P. falciparum* parasites with no *hrp2/3* deletion, *hrp2* deletion, *hrp3* deletion, and *hrp2/3* deletion are shown for Sudan and the 2 study sites, Al Jazirah and Al Qadarif.

## Conclusions

The spread of the *Pfkelch13* 622I mutant in the Horn of Africa is alarming because it raises the potential for ART-R to take hold in this region. Data from this study show parallels with findings from Eritrea (*8*) and possibly Ethiopia (*10*). With confirmed cases in Eritrea and possible cases in Ethiopia (*8*,*10*), the presence of this mutant in Sudan suggests a possible cross-border spread that could threaten malaria treatment in several countries. If left uncontained, *Pfkelch13* 622I mutant parasites could undermine the efficacy of ACTs, threatening progress in malaria control in East Africa. Rapid regional surveillance and coordinated response efforts are urgently needed to contain this threat before it escalates further. In addition, the presence of *hrp2/hrp3* gene deletions in 10.7% of the *Pfkelch13* 622I mutants reported in this study is an additional concern, because it affects the results of HRP2-based RDTs in detecting *P. falciparum* parasites. Because of the presence of those gene deletions, the variants could spread more easily because they are not detected by the HRP2-based RDT, complicating malaria control efforts in affected regions.

The first limitation of this study is that we could not confirm the association between the presence of *Pfkelch13* 622I and the persistence of parasitemia on day 3, because clinical data were not available. Second, our data came from only 2 health facilities with limited catchment areas. Third, the samples were collected in 2017, but the findings remain highly relevant in 2025 because they provide critical baseline data on the early spread of resistance markers in Sudan. Although clinical reports of treatment failures in Sudan remain limited, our data are essential for understanding the persistence and regional transmission dynamics of ART-R *P. falciparum* parasites, which is crucial for guiding current malaria control strategies.

Our findings increase the need for further studies to determine whether the *Pfkelch13* 622I mutants observed in Sudan represent a local emergence or are directly linked to the *P. falciparum* parasite population from Eritrea or Ethiopia, as well as to clarify the flow of the *Pfkelch13* 622I mutant parasite population in this region. With ART-R now confirmed in *P. falciparum* and *hrp2* and *hrp3* deletions in parasite populations in Sudan, additional strategies must be implemented to contain the spread of these lineages across the Horn of Africa. Otherwise, the emergence of partner drug resistance could lead to higher rates of treatment failure and uncontrolled spread of potentially resistant *P. falciparum* parasites beyond this region.

Appendix Additional information about high prevalence of artemisinin-resistant *Plasmodium falciparum*, southeastern Sudan.

## References

[1] World Health Organization. World malarial report 2024: addressing inequity in the global malaria response. [cited 2025 Feb 4]. https://www.who.int/teams/global-malaria-programme/reports/world-malaria-report-2024

[2] Ariey F, Witkowski B, Amaratunga C, Beghain J, Langlois AC, Khim N, et al. A molecular marker of artemisinin-resistant *Plasmodium falciparum* malaria. Nature. 2014;505:50–5. 10.1038/nature12876 24352242 PMC5007947

[3] Ashley EA, Dhorda M, Fairhurst RM, Amaratunga C, Lim P, Suon S, et al.; Tracking Resistance to Artemisinin Collaboration (TRAC). Spread of artemisinin resistance in *Plasmodium falciparum* malaria. N Engl J Med. 2014;371:411–23. 10.1056/NEJMoa1314981 25075834 PMC4143591

[4] Dondorp AM, Nosten F, Yi P, Das D, Phyo AP, Tarning J, et al. Artemisinin resistance in *Plasmodium falciparum* malaria. N Engl J Med. 2009;361:455–67. 10.1056/NEJMoa0808859 19641202 PMC3495232

[5] Conrad MD, Asua V, Garg S, Giesbrecht D, Niaré K, Smith S, et al. Evolution of partial resistance to artemisinins in malaria parasites in Uganda. N Engl J Med. 2023;389:722–32. 10.1056/NEJMoa2211803 37611122 PMC10513755

[6] Ishengoma DS, Mandara CI, Bakari C, Fola AA, Madebe RA, Seth MD, et al. Evidence of artemisinin partial resistance in northwestern Tanzania: clinical and molecular markers of resistance. Lancet Infect Dis. 2024;24:1225–33. 10.1016/S1473-3099(24)00362-1 39159633 PMC11511676

[7] Juliano JJ, Giesbrecht DJ, Simkin A, Fola AA, Lyimo BM, Pereus D, et al. Prevalence of mutations associated with artemisinin partial resistance and sulfadoxine-pyrimethamine resistance in 13 regions in Tanzania in 2021: a cross-sectional survey. Lancet Microbe. 2024;5:100920. 10.1016/S2666-5247(24)00160-5 39159629 PMC11464622

[8] Mihreteab S, Platon L, Berhane A, Stokes BH, Warsame M, Campagne P, et al. Increasing prevalence of artemisinin-resistant HRP2-negative malaria in Eritrea. N Engl J Med. 2023;389:1191–202. 10.1056/NEJMoa2210956 37754284 PMC10539021

[9] Uwimana A, Legrand E, Stokes BH, Ndikumana JM, Warsame M, Umulisa N, et al. Emergence and clonal expansion of in vitro artemisinin-resistant *Plasmodium falciparum* kelch13 R561H mutant parasites in Rwanda. Nat Med. 2020;26:1602–8. 10.1038/s41591-020-1005-2 32747827 PMC7541349

[10] Fola AA, Feleke SM, Mohammed H, Brhane BG, Hennelly CM, Assefa A, et al. *Plasmodium falciparum* resistant to artemisinin and diagnostics have emerged in Ethiopia. Nat Microbiol. 2023;8:1911–9. 10.1038/s41564-023-01461-4 37640962 PMC10522486

[11] World Health Organization Eastern Mediterranean Region. Sudan’s adoption of “high burden to high impact” approach to boost malaria control efforts. 2022 [cited 2025 Feb 4]. https://www.emro.who.int/malaria/rbm-news/sudans-adoption-of-high-burden-to-high-impact-approach-to-boost-malaria-control-efforts.html

[12] Prosser C, Gresty K, Ellis J, Meyer W, Anderson K, Lee R, et al. *Plasmodium falciparum* histidine-rich protein 2 and 3 gene deletions in strains from Nigeria, Sudan, and South Sudan. Emerg Infect Dis. 2021;27:471–9. 10.3201/eid2702.191410 33496220 PMC7853540

[13] Elagali A, Ahmed A, Makki N, Ismail H, Ajak M, Alene KA, et al. Spatiotemporal mapping of malaria incidence in Sudan using routine surveillance data. Sci Rep. 2022;12:14114. 10.1038/s41598-022-16706-1 35982088 PMC9387890

[14] Zainabadi K, Adams M, Han ZY, Lwin HW, Han KT, Ouattara A, et al. A novel method for extracting nucleic acids from dried blood spots for ultrasensitive detection of low-density *Plasmodium falciparum* and *Plasmodium vivax* infections. Malar J. 2017;16:377. 10.1186/s12936-017-2025-3 28923054 PMC5604154

