# Correction: Vol. 31, No. 6

**DOI:** 10.3201/eid3108.C33108

**Published:** 2025-08

**Keywords:** errata, erratum, correction

A category label was incorrect in Table 1 of High Prevalence of Artemisinin-Resistant Plasmodium falciparum, Southeastern Sudan (M. L’Episcopia et al.). The article has been corrected online (https://wwwnc.cdc.gov/eid/article/31/6/24-1810_article).

