# Supplementary material for: High Prevalence of Artemisinin-Resistant Plasmodium falciparum, Southeastern Sudan
Source: Emerg Infect Dis. 2025 Jun;31(6):1211–5. doi: 10.3201/eid3106.241810 (PMC12123913; doi:10.3201/eid3106.241810)
Supplement: Appendix — Additional information about high prevalence of artemisinin-resistant Plasmodium falciparum, southeastern Sudan. [file 24-1810-Techapp-s1.pdf]

Article DOI: https://doi.org/10.3201/eid3106.241810

EID cannot ensure accessibility for supplementary materials supplied by authors. Readers who have difficulty accessing supplementary content should contact the authors for assistance.

High Prevalence of Artemisinin-Resistant *Plasmodium falciparum*, Southeastern Sudan

Appendix

***Plasmodium falciparum* malaria in Sudan.** Sudan is a major contributor to malaria in the WHO Eastern Mediterranean Region, accounting for 33% of estimated malaria cases and 44% of estimated deaths (*1*,*2*). *Plasmodium falciparum* is the predominant malaria parasite in Sudan, responsible for approximately 95% of malaria cases (*3*). Malaria transmission in the country is characterized as low to moderate, highly seasonal and occasionally epidemic (*3*).

From 2017 to 2019, Sudan experienced high malaria incidence, with regional variability and fluctuations driven by environmental and epidemiological factors. The situation has been exacerbated by armed conflict, political instability and economic disruption, leading to a collapse of the health system and an increased burden of malaria (*1*). In response to a more than 40% increase in malaria incidence (*4*), Sudan adopted the High Burden to High Impact (HBHI) strategy in 2022 (*1*,*4*). However, despite interventions, several biological threats continue to challenge malaria control efforts. These include *P. falciparum* resistance to antimalarial drugs (*5–7*) and insecticides (*8–10*), *pfhrp2/3* gene deletions affecting the performance of rapid diagnostic tests (*11*,*12*), and the recent invasion of *Anopheles stephensi*, a highly adaptable urban malaria vector (*13*).

Efforts to map malaria incidence using spatio-temporal methods have provided insights into its spread and intensity (*3*). Nevertheless, the emergence of drug-resistant malaria strains and vector adaptation remain major challenges.

***Plasmodium falciparum* antimalarial drug resistance in Sudan.** Sudan has faced challenges with antimalarial drug resistance, leading to changes in treatment policies.

Chloroquine resistance emerged in the late 1970s (*14*), prompting a switch to artemisinin-based combination therapies (ACTs) in 2004-2006 (*15*). Studies have shown high prevalence of mutations in drug resistance genes, particularly in *Pfcrt, Pfmdr-1, dhfr*, and *dhps* (*15*,*16*). The *Pfcrt-76T* and *Pfmdr1-86Y* mutations associated with chloroquine resistance have decreased, while *dhfr* and *dhps* mutations linked to sulfadoxine-pyrimethamine resistance have increased (*15*). No *Pfkelch13* mutations associated with artemisinin partial resistance (ART-R) were detected in central and eastern Sudan (*15*,*17*). However, novel *Pfkelch13* mutations not linked to ART-R (F375S, K378R, D389N, K430K, E433D, P443P, N594K) were observed in the Blue Nile State (*17*).

Antimalarial drug efficacy studies in Sudan between 2004 and 2018 have shown varying results for different treatments. Chloroquine and sulfadoxine-pyrimethamine monotherapies demonstrated high failure rates, necessitating a shift to artemisinin-based combination therapies (ACTs) (*18*). Artesunate plus SP (AS+SP) and artemether-lumefantrine (AL) became first-line and second-line treatments, respectively (*14*). While AL maintained high efficacy (96.8-100%), AS+SP showed declining effectiveness in some regions, particularly eastern Sudan (*14*). A meta-analysis revealed overall high treatment success (98%) for ACTs, with AL slightly outperforming AS+SP (*5*). However, a comparative study in eastern Sudan found AS+SP to have marginally higher efficacy than AL after PCR correction (*19*). As reported by Adam et al., in 2023 (*6*) high efficacy rates (97%) of AL was confirmed in Sudan, except for Sennar site in the 2017–2018 where a lower cure rate (92.1%) was detected which was still above the 90% threshold for treatment policy change (*20*).

Supplementary materials and Methods

Screening and recruitment of study participants. Eligible patients were at least one year of age, presenting with suspected uncomplicated *P. falciparum* infection with fever (axillary temperature ≥37.5°C) and/or history of fever in the past 24h.

Enrolled patients were assigned to receive a supervised standard 3-day course of artemether-lumefantrine AL (IPCA 20 mg artemether and 120 mg lumefantrine per tablet; 6 tablets for 5kg to <15kg; 12 tablets for 15 to <25kg; 18 tablets for 25 to <35kg; 24 tablets for ≥35kg) in 2017 according to the manufacturer's dosing schedule.

Informed written consent was obtained from the adult patient or parent/caretaker of a child prior to recruitment. If a patient or parent/caretaker was illiterate, consent was obtained in the presence of a witness (witness' signature and the thumbprint of the participant's parent/caretaker). Children aged 12 years and above also signed informed assents in addition to their parents' consent. The study was approved by the relevant committees of the Ministry of Health in Gezira State, which serves as the central committee in Sudan (MU/2019).

Sample collection. Patients were monitored clinically and parasitologically at recruitment. Thick and thin smears were obtained by finger prick prior to the initiation of treatment and blood were spotted onto filter papers for molecular studies. Patients were advised to come to the health facilities for unscheduled visits if symptoms developed.

DNA extraction and molecular analysis. Parasite DNA was extracted from dried blood spots (DBS) collected pretreatment according to the Zainabadi et al. protocol (*21*).

Molecular signatures associated with antimalarial drug resistance. Parasite DNA extracted from blood samples collected on day 0 was analyzed for molecular markers associated with antimalarial drug resistance. We assessed point mutations in the *Pfkelch13* propeller domain (codons 430–720), which are linked to artemisinin partial resistance (ART-R). Additionally, we examined mutations in the following genes associated with resistance to different antimalarial drugs: *Pfcrt* (codons 72–76, 93, 97, 145, 218, 343, 350, 353, and 356) – associated with 4-aminoquinoline resistance; *Pfmdr1* (codons 86, 184, 1034, 1042, and 1246) – associated with amino-alcohol resistance; *dhfr* (codons 16, 50, 51, 59, 108, and 164) – associated with pyrimethamine resistance; *dhps* (codons 431, 436, 437, 540, 581, and 613) – associated with sulfadoxine resistance. We also tested for deletions in *hrp2* and *hrp3*, which can impact the performance of HRP2-based malaria rapid diagnostic tests (RDTs) (*22*).

PCR Amplification and Sequencing Protocol. Amplicons from the targeted regions (presented in the table below) were generated using multiplex nested PCR assays with indexed primers containing specific barcodes. A total of 4 μL of PCR products from each sample was pooled (96 samples per pool) to optimize sample usage in downstream steps. Each pool underwent AMPure XP bead purification (Beckman Coulter Life Sciences, Villepinte, France) following the manufacturer's protocol to remove dNTPs, salts, primers, and primer dimers. The purified amplicons were then analyzed on a Fragment Analyzer (Agilent, Agilent Technologies,

Les Ulis, France) to assess DNA quality, and DNA concentrations were measured using fluorometric quantitation (Qubit, Thermo Fisher, Scientific, Illkirch-Graffenstaden, France). The pooled libraries were denatured with NaOH (0.1 N final concentration) and diluted with hybridization buffer before sequencing. Sequencing was performed using the MiSeq v2 reagent kit (300-cycle, Illumina Inc., Evry-Courcouronnes, France), following the manufacturer's recommendations (*22*).

Appendix Table 1. List of the targeted regions and number of amplicons generated by multiplexing nested PCR assays

Gene Name	Gene ID	Chr.	Gene location	SNPs/CNVs detection	No. of amplicons
Pfkelch13	PF3D7_1343700	13	1,724,817 - 1,726,997 (-)	SNP from codon 440 to 727	12
chloroquine resistance transporter	PF3D7_0709000	7	403,222 - 406,317 (+)	Codons 72-76, 93, 97, 145, 218, 343, 350, 353	5
multidrug resistance protein 1	PF3D7_0523000	5	957,890 - 962,149 (+)	Codons 86, 184, 1034, 1042, 1246	5
bifunctional dihydrofolate reductase-thymidylate synthase	PF3D7_0417200	4	748,088 - 749,914 (+)	Codons 16, 50, 51, 59, 108, 164	5
hydroxymethyldihydropterin pyrophosphokinase-dihydropteroate synthase	PF3D7_0810800	8	548,200 - 550,616 (+)	Codons 436, 437, 540, 581, 613	4
histidine-rich protein 2	PF3D7_0831800	8	1,373,212 – 1,376,988 (-)	Deletion	2
histidine-rich protein 3	PF3D7_1372200	13	2,840,236 – 2,842,840 (-)	Deletion	2

Bioinformatics Processing and Variant Calling. Raw sequencing reads were demultiplexed and quality-trimmed at a Phred score threshold of 30 to ensure high-quality data. Primer sequences were removed from the 5′-end of the reads to eliminate potential primer bias in downstream analyses. Base calling was performed by aligning reads against a custom database, which included the *Plasmodium falciparum* 3D7 reference genome (v45). Bioinformatic analyses were conducted using CLC Genomics Workbench 25 (Qiagen, Courtaboeuf, France) for sequence alignment, variant calling, and quality assessment. To ensure accuracy and reliability, laboratory reference *P. falciparum* strains with known alleles were included as controls. These strains comprised Dd2, 7G8, HB3, and 3601 (a Cambodian culture-adapted parasite), as detailed in the table below (*22*).

Appendix Table 2. List of the alleles of the parasite strains used as controls

Gene name	Dd2 allele	7G8 allele	HB3 allele	3601 strain
Pfkelch13	Wild-type	Wild-type	Wild-type	C580Y
chloroquine resistance transporter	CVIETTHFIMCGT	SVMNTTHFIMCGT	CVMNKTHFIMCGI	CVIETTHFIMCGT
multidrug resistance protein 1	YYSND	NFCDY	NFSDD	NFSND
bifunctional dihydrofolate reductase-thymidylate synthase	ACIRNI	ACICNI	ACNCNI	ACIRNI
hydroxymethyldihydropterin pyrophosphokinase-dihydropteroate synthase	IFGKAS	ISGKAA	ISAKAA	IAGEAA
histidine-rich protein 2	Deletion	NA	NA	NA
histidine-rich protein 3	NA	NA	Deletion	NA

Position of amino acid corresponds to codons 72, 73, 74, 76, 93, 97, 145, 218, 343, 350, 353 and 356 for pfcrt, to codons 86, 184, 1034, 1042 and 1246 for pfmdr-1, to codons 16, 50, 51, 59, 108, 164 for dhfr and to codons 431, 436, 437, 540, 581, 613 for dhps.

References

1. World Health Organization. World malarial report 2024: addressing inequity in the global malaria response. [cited 2025 Feb 4]. https://www.who.int/teams/global-malaria-programme/reports/world-malaria-report-2024

2. Singh Pardal MPR, Orkeh GO. Overview of malaria situation in Sudan. Int J Community Med Public Health. 2020;7:1721–5. https://doi.org/10.18203/2394-6040.ijcmph20201970

3. Elagali A, Ahmed A, Makki N, Ismail H, Ajak M, Alene KA, et al. Spatiotemporal mapping of malaria incidence in Sudan using routine surveillance data. Sci Rep. 2022;12:14114. PubMed https://doi.org/10.1038/s41598-022-16706-1

4. World Health Organization Eastern Mediterranean Region. Sudan's adoption of "high burden to high impact" approach to boost malaria control efforts. 2022 [cited 2025 Feb 4]. https://www.emro.who.int/malaria/rbm-news/sudans-adoption-of-high-burden-to-high-impact-approach-to-boost-malaria-control-efforts.html

5. Adam I, Ibrahim Y, Gasim GI. Efficacy and safety of artemisinin-based combination therapy for uncomplicated *Plasmodium falciparum* malaria in Sudan: a systematic review and meta-analysis. Malar J. 2018;17:110. PubMed https://doi.org/10.1186/s12936-018-2265-x

6. Adam M, Nahzat S, Kakar Q, Assada M, Witkowski B, Tag Eldin Elshafie A, et al. Antimalarial drug efficacy and resistance in malaria-endemic countries in HANMAT-PIAM_net countries of the eastern Mediterranean region 2016–2020: clinical and genetic studies. Trop Med Int Health. 2023;28:817–29. PubMed https://doi.org/10.1111/tmi.13929

7. Hussien M, Abdel Hamid MM, Elamin EA, Hassan AO, Elaagip AH, Salama AHA, et al. Antimalarial drug resistance molecular makers of *Plasmodium falciparum* isolates from Sudan during 2015–2017. PLoS One. 2020;15:e0235401. PubMed https://doi.org/10.1371/journal.pone.0235401

8. Ismail BA, Kafy HT, Sulieman JE, Subramaniam K, Thomas B, Mnzava A, et al. Temporal and spatial trends in insecticide resistance in *Anopheles arabiensis* in Sudan: outcomes from an evaluation of implications of insecticide resistance for malaria vector control. Parasit Vectors. 2018;11:122. PubMed https://doi.org/10.1186/s13071-018-2732-9

9. Kafy HT, Ismail BA, Mnzava AP, Lines J, Abdin MSE, Eltaher JS, et al. Impact of insecticide resistance in *Anopheles arabiensis* on malaria incidence and prevalence in Sudan and the costs of mitigation. Proc Natl Acad Sci U S A. 2017;114:E11267–75. PubMed https://doi.org/10.1073/pnas.1713814114

10. Korti MY, Ageep TB, Adam AI, Shitta KB, Hassan AA, Algadam AA, et al. Status of insecticide susceptibility in *Anopheles arabiensis* and detection of the knockdown resistance mutation (kdr) concerning agricultural practices from northern Sudan state, Sudan. J Genet Eng Biotechnol. 2021;19:49. PubMed https://doi.org/10.1186/s43141-021-00142-1

11. Lynch E, Jensen TO, Assao B, Chihana M, Turuho T, Nyehangane D, et al. Evaluation of HRP2 and pLDH-based rapid diagnostic tests for malaria and prevalence of pfhrp2/3 deletions in Aweil, south Sudan. Malar J. 2022;21:261. PubMed https://doi.org/10.1186/s12936-022-04280-w

12. Molina-de la Fuente I, Benito MJS, Flevaud L, Ousley J, Pasquale HA, Julla A, et al. *Plasmodium falciparum* pfhrp2 and pfhrp3 gene deletions in malaria-hyperendemic region, south Sudan. Emerg Infect Dis. 2023;29:154–9. PubMed https://doi.org/10.3201/eid2901.220775

13. Ahmed A, Khogali R, Elnour MB, Nakao R, Salim B. Emergence of the invasive malaria vector *Anopheles stephensi* in Khartoum State, central Sudan. Parasit Vectors. 2021;14:511. PubMed https://doi.org/10.1186/s13071-021-05026-4

14. Adeel AA. Drug-resistant malaria in Sudan: a review of evidence and scenarios for the future. Sudan J Paediatr. 2012;12:8–20. PubMed

15. Bakhiet AMA, Abdelraheem MH, Kheir A, Omer S, Gismelseed L, Abdel-Muhsin AA, et al. Evolution of *Plasmodium falciparum* drug resistance genes following artemisinin combination therapy in Sudan. Trans R Soc Trop Med Hyg. 2019;113:693–700. PubMed https://doi.org/10.1093/trstmh/trz059

16. L'Episcopia M, Kelley J, Patel D, Schmedes S, Ravishankar S, Menegon M, et al. Targeted deep amplicon sequencing of kelch 13 and cytochrome b in *Plasmodium falciparum* isolates from an endemic African country using the malaria resistance surveillance (MaRS) protocol. Parasit Vectors. 2020;13:137. PubMed https://doi.org/10.1186/s13071-020-4005-7

17. Mohamed AO, Hussien M, Mohamed A, Suliman A, Elkando NS, Abdelbagi H, et al. Assessment of *Plasmodium falciparum* drug resistance molecular markers from the Blue Nile State, southeast Sudan. Malar J. 2020;19:78. PubMed https://doi.org/10.1186/s12936-020-03165-0

18. Hamour S, Melaku Y, Keus K, Wambugu J, Atkin S, Montgomery J, et al. Malaria in the Nuba Mountains of Sudan: baseline genotypic resistance and efficacy of the artesunate plus sulfadoxine-pyrimethamine and artesunate plus amodiaquine combinations. Trans R Soc Trop Med Hyg. 2005;99:548–54. PubMed https://doi.org/10.1016/j.trstmh.2004.10.003

19. Mukhtar EA, Gadalla NB, El-Zaki SE, Mukhtar I, Mansour FA, Babiker A, et al. A comparative study on the efficacy of artesunate plus sulphadoxine/pyrimethamine versus artemether-lumefantrine in eastern Sudan. Malar J. 2007;6:92. PubMed https://doi.org/10.1186/1475-2875-6-92

20. World Health Organization. Report on antimalarial drug efficacy, resistance and response: 10 years of surveillance (2010–2019). Nov 2020. [cited 2025 Feb 4]. https://www.who.int/publications/i/item/9789240012813

21. Zainabadi K, Adams M, Han ZY, Lwin HW, Han KT, Ouattara A, et al. A novel method for extracting nucleic acids from dried blood spots for ultrasensitive detection of low-density *Plasmodium falciparum* and *Plasmodium vivax* infections. Malar J. 2017;16:377. PubMed https://doi.org/10.1186/s12936-017-2025-3

22. Mihreteab S, Platon L, Berhane A, Stokes BH, Warsame M, Campagne P, et al. Increasing prevalence of artemisinin-resistant hrp2-negative malaria in Eritrea. N Engl J Med. 2023;389:1191–202. PubMed https://doi.org/10.1056/NEJMoa2210956
